# Embracing polygenicity: a review of methods and tools for psychiatric genetics research

**DOI:** 10.1017/S0033291717002318

**Published:** 2017-08-29

**Authors:** R. M. Maier, P. M. Visscher, M. R. Robinson, N. R. Wray

**Affiliations:** 1 Queensland Brain Institute, University of Queensland, Brisbane, Queensland, Australia; 2 Institute for Molecular Bioscience, University of Queensland, Brisbane, Queensland, Australia; 3 Department of Computational Biology, University of Lausanne, Lausanne, Switzerland

**Keywords:** Genetics, methods, polygenic, review

## Abstract

The availability of genome-wide genetic data on hundreds of thousands of people has led to an equally rapid growth in methodologies available to analyse these data. While the motivation for undertaking genome-wide association studies (GWAS) is identification of genetic markers associated with complex traits, once generated these data can be used for many other analyses. GWAS have demonstrated that complex traits exhibit a highly polygenic genetic architecture, often with shared genetic risk factors across traits. New methods to analyse data from GWAS are increasingly being used to address a diverse set of questions about the aetiology of complex traits and diseases, including psychiatric disorders. Here, we give an overview of some of these methods and present examples of how they have contributed to our understanding of psychiatric disorders. We consider: (i) estimation of the extent of genetic influence on traits, (ii) uncovering of shared genetic control between traits, (iii) predictions of genetic risk for individuals, (iv) uncovering of causal relationships between traits, (v) identifying causal single-nucleotide polymorphisms and genes or (vi) the detection of genetic heterogeneity. This classification helps organise the large number of recently developed methods, although some could be placed in more than one category. While some methods require GWAS data on individual people, others simply use GWAS summary statistics data, allowing novel well-powered analyses to be conducted at a low computational burden.

## Introduction

The reduction in costs of genotyping technologies in recent years has led to an explosion of genetic and phenotypic information collected on large numbers of people. The primary aim of these studies is to identify genetic polymorphisms associated with a quantitative trait or with an increased risk of disease. These genome-wide association studies (GWAS) have led to an important increase in understanding of the underpinnings of psychiatric and other disorders (Sullivan *et al.* 2012; Visscher *et al.* 2012; Gratten, 2016). They have provided empirical data demonstrating that common disorders are polygenic and have allowed interrogation of the genetic architecture, in terms of number, frequency, effect size and interactions of genetic risk factors. However, the potential use of GWAS data goes far beyond identification of trait associated loci.

Here, we present an overview of some recently developed methods utilising genome-wide genotype and phenotype data on large numbers of individuals and show how they can be applied to the research of psychiatric disorders. These new methods serve at least one of the following purposes: (i) estimation of the extent of genetic influence on traits (Estimation of proportion of variance attributable to genome-wide single-nucleotide polymorphisms (SNPs), SNP-heritability or *h*^2^_SNP_), (ii) uncovering of shared genetic control between traits (Estimation of genetic correlation from using genome-wide SNPs), (iii) predictions of genetic risk for individuals (Polygenic risk prediction), (iv) uncovering of causal relationships between traits [Mendelian randomisation (MR)], (v) identifying causal SNPs and genes (Fine-mapping and gene prioritisation), or (vi) Detection of genetic heterogeneity. There is often an overlap between these applications: some methods can be applied for more than one purpose, and some of the available software implements more than one method. In this overview, it is not possible to be exhaustive, and we apologise to authors whose methods have not been included.

Most of the methods presented here require genetic data in either of two formats. One is that of full individual level genotype data and phenotypic measurements on each person, where the genetic data can be represented as a matrix with allele counts for each genetic marker for each person. While this offers the largest range of analytic options, file sizes can be very large, which can become prohibitive as computational burden is usually non-linear with increasing numbers of individuals and markers. Moreover, privacy concerns can prevent this type of data from being shared across research groups. Summary statistics of genome-wide association analysis represent the second data format, for which data sharing has fewer privacy concerns (Pasaniuc & Price, 2016). GWAS summary statistics comprise the association test statistic (including direction of effect for a reference allele), standard error, *p* value of association and allele frequency of each SNP. While it has been shown that it is possible to infer whether an individual was part of a cohort using summary statistics, the power to do so is limited (Homer *et al.* 2008; Sankararaman *et al.* 2009; Visscher & Hill, 2009), and in any case requires the genome-wide genotype data of the individual to be identified. To guard against any privacy concerns GWAS summary statistics can be provided using allele frequencies estimated in large independent samples of the same ethnicity. Methods which require only summary statistic data benefit from shorter analytical run-times, much reduced computer memory requirements, and applicability to a larger number of traits. These benefits usually come at the cost of lower precision (larger standard errors), when compared with methods that utilise individual level genotype data.

While genetic data in one of these two formats are required by all methods presented here, some methods additionally make use of other information, such as genomic annotation or expression quantitative trait locus (eQTL) data. Genomic annotation can give clues about the functional importance of a region in which a SNP resides, whereas eQTL data are the result of an association test where the phenotype analysed is expression of a gene.

**Fig. 1.**
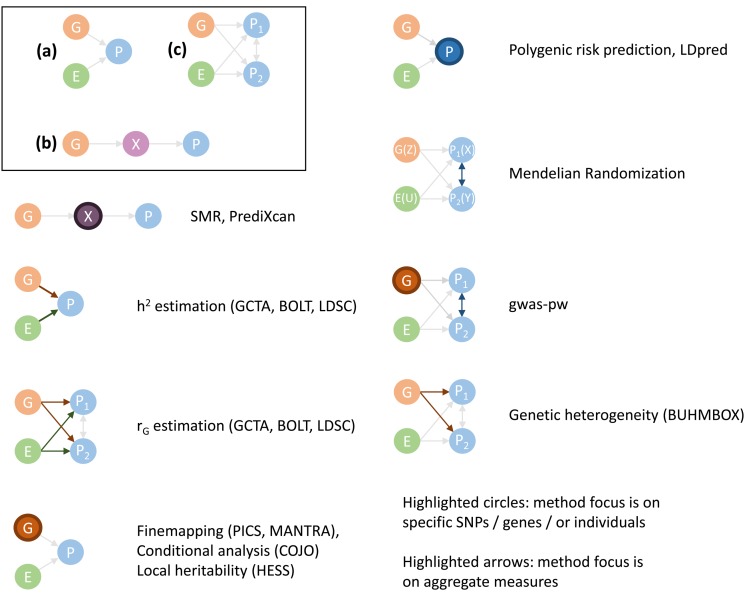
Schematic representation of the basic models underlying the polygenic methods reviewed. All models assume that a phenotype (**P**) is influenced by genetic (**G**) and environmental (**E**) factors (with environmental defined loosely as anything not captured by G including stochastic variation and measurement error). (*a*) Model which considers only one phenotype and no gene expression. (*b*) Model which considers only one phenotype and gene expression (**X**). (*c*) Model which considers two or more phenotypes and no gene expression. Methods can be grouped into those where the focus lies on individual SNPs, genes or people (nodes highlighted), and those where the focus lies on aggregate measures affecting the relationship between genetic and environmental factors and a phenotype (edges highlighted).

Here we review a range of different polygenic methods and highlight their aims and the input data they require (Fig. 1). For each method, we provide some examples of applications relevant to psychiatric genetics research (Table 1).

**Table 1.** Overview of selected polygenic methods

Program/method	Data needed	URL
Estimation of *h*^2^, *r*_G_		
GCTA (GREML)	Individual-level genotype data	http://cnsgenomics.com/software/gcta/
BOLT-REML/BOLT-LMM	Individual-level genotype data	https://data.broadinstitute.org/alkesgroup/BOLT-LMM/
LD score regression	Summary statistics + LD scores	https://github.com/bulik/ldsc; http://ldsc.broadinstitute.org/
HESS (local heritability)	Summary statistics + LD reference panel	http://bogdan.bioinformatics.ucla.edu/software/hess
Polygenic risk prediction		
PLINK	Summary statistics + individual-level data	https://www.cog-genomics.org/plink2
PRSice	Summary statistics + individual-level data	http://prsice.info/
GCTA, MTG2 (GBLUP, MTGBLUP)	Individual-level genotype data	http://cnsgenomics.com/software/gcta/; https://sites.google.com/site/honglee0707/mtg2
BayesR	Individual-level genotype data	https://github.com/syntheke/bayesR
LDpred	Summary statistics + individual-level data	https://github.com/bvilhjal/ldpred
Causality of phenotypes		
Mendelian randomisation	Individual-level genotype data or summary statistics	http://www.mrbase.org/
gwas-pw	Summary statistics	https://github.com/joepickrell/gwas-pw
Causality of genes (gene prioritisation)		
gwas-pw	Summary statistics	https://github.com/joepickrell/gwas-pw
SMR	Summary statistics + eQTL	http://cnsgenomics.com/software/smr/
PrediXcan	Individual-level genotype data + eQTL	https://github.com/hakyimlab/PrediXcan
metaXcan	Summary statistics + eQTL	https://github.com/hakyimlab/MetaXcan
TWAS/FUSION	Summary statistics + eQTL	http://gusevlab.org/projects/fusion/
DEPICT	Summary statistics	https://data.broadinstitute.org/mpg/depict/
Causality of SNPs (fine-mapping)		
PICS (Fine-mapping)	Summary statistics	http://pubs.broadinstitute.org/pubs/finemapping/
GCTA (COJO)	Summary statistics	http://cnsgenomics.com/software/gcta/
MANTRA	Summary statistics	Available on request from the author
Detection of genetic heterogeneity		
BUHMBOX	Summary statistics + individual-level data	http://software.broadinstitute.org/mpg/buhmbox/
Subtest	Summary statistics	https://github.com/jamesliley/subtest

## Estimation of proportion of variance attributable to genome-wide SNPs

Heritability is the proportion of phenotypic variance that can be attributed to genetic factors. It is a key quantity in genetics research as it summarises the role of causal inherited variation. Trait heritability can be estimated by comparing the phenotypic resemblance among family members to their coefficients of relationships. However, estimating heritability from relatives can result in upwards-biased estimates (Vinkhuyzen *et al.* 2013) if non-genetic factors shared by relatives cannot be disentangled from the shared genetic relationships. This can be circumvented by estimating heritability from genome-wide markers in unrelated (that is, very distantly related) individuals. Genomic-restricted maximum-likelihood analysis (GREML) can be used to estimate the proportion of phenotypic variance, which is captured by genotyped SNPs (SNP-heritability), by using genetic data of unrelated individuals (Yang *et al.* 2010). SNP-heritability estimates are typically lower than those from twin or family-based heritability estimates, because genotyped SNPs account only for a subset of all genetic effects (the remainder includes other types of polymorphisms and SNPs that are not tagged by genotyped SNPs). Hence, the parameter estimated by SNP-heritability analysis depends on the genotype data available in the data set analysed, and can only converge to the traditional parameter being estimated from family data, when the genotypes available are fully representative of the variation in the genome.

Estimation of SNP-heritability has been of particular importance for disease traits, especially those of low lifetime risk (<1% is typical of most common diseases) for which it is difficult to collect the large samples needed to calculate heritability from estimates of increased risk in relatives of those affected. Both traditional-heritability and SNP-heritability estimates are presented on the liability scale (and depend on lifetime risk of disease in the population), and empirical data of the GWAS era (Lee *et al.* 2012*a*; Stahl *et al.* 2012) demonstrates that the polygenic model implied in these estimates is justified. GWAS case–control samples for disease traits are usually heavily oversampled for cases compared with a population sample and so SNP-heritability estimates are made on this binary case–control scale and transformed to the liability scale accounting for this ascertainment (Lee *et al.* 2011). The SNP-heritability estimates are relatively robust to choice of lifetime risk for most common diseases (lifetime risk <1%). However, for the very common diseases such as major depressive disorder (MDD) (lifetime risk 15%), SNP-heritability estimates are more sensitive to the choice of lifetime risk estimate, and to screening *v.* non-screening of controls (Peyrot *et al.* 2016).

The GREML method for estimation of SNP-heritability is based on a linear mixed model (Meuwissen *et al.* 2001), where a central component is the genetic relationship (or similarity) matrix, which captures the genetic relatedness of all pairs of individuals (Hayes *et al.* 2009). For application to human data, the algorithm is implemented in GCTA (Yang *et al.* 2011*a*). Several independent studies have confirmed that GREML results in unbiased estimates of SNP heritability when the model assumptions are met, or have investigated how departures from these assumptions can influence the results (Speed *et al.* 2012; Zhou & Stephens, 2014). Potential biases that might influence heritability estimation are an association between minor allele frequency (MAF) and SNP effect size, which does not fit the assumptions of the model, or an overrepresentation of causal SNPs in regions of high or low linkage disequilibrium (LD) (Lee *et al.* 2011; Speed *et al.* 2012), both of which can be overcome in GCTA through the use of the GREML–LDMS approach (Yang *et al.* 2015*a*). The runtime of GCTA–GREML is a function of both the number of markers, *M*, and the number of individuals, *N*. Compute time is *O*(*N*^3^ + *MN*^2^), which includes a component for the construction of the genetic relationship matrix and a component for the actual REML algorithm. The steep increase in runtime with larger *N* makes it impractical for data sets with very large numbers of individuals. The software BOLT-LMM can estimate variance components through a stochastic approximation algorithm, which circumvents the costly calculation of a genetic relationship matrix and thus reduces the runtime to only *O*(*MN*^1.5^) (Loh *et al.* 2015). Note that both GCTA and BOLT-LMM have other features such as linear mixed model association analysis (Yang *et al.* 2014) and genotype × environment (G × E) analysis, which are not the topic of this review.

LD score regression (LDSC) (Bulik-Sullivan *et al.* 2015*b*) is a method, which requires only summary statistics to estimate SNP-heritability and has therefore shorter runtime than the methods discussed so far. Under polygenic genetic architecture, SNPs which are highly correlated with many other SNPs (have a high LD score) are more likely to tag a causal SNP and are therefore expected, on average, to have a higher association test statistic than SNPs which are not highly correlated with many other SNPs. The regression coefficient of the association test statistics of all SNPs on their LD score is a function of SNP-heritability (Yang *et al.* 2011*b*; Bulik-Sullivan *et al.* 2015*b*). Any factor that increases the association statistic of a SNP independently of its LD score (as might be found in population stratification, which induces correlations in test statistics across chromosomes) will increase the intercept term of this regression (Bulik-Sullivan *et al.* 2015*b*). LDSC estimates SNP-heritability with vastly reduced computational speed compared with GREML, but with higher standard error (s.e.) of estimates. The s.e. of GREML SNP-heritability estimates is accurately approximated as 316/*N*, where *N* is the total sample size (Visscher *et al.* 2014). For LDSC the standard errors of the variance component estimates are typically larger (usually by 50% or more) than those of a GREML analysis for the same sample size (Yang *et al.* 2015*b*). However, it is typical that LDSC can be applied to larger data sets (which generate smaller s.e.) since only summary statistics are needed. Comparisons of estimates from GREML and LDSC show that the accuracy of estimates from LDSC are dependent on LD scores calculated from a population representative of the population used to estimate GWAS summary statistics (Yang *et al.* 2015*a*; Brown *et al.* 2016).

GREML, LDSC and similar methods can also be used to estimate the SNP-heritability based on genomic partitioning, for example by chromosome, minor allele frequency, genomic annotations or single loci (Shi *et al.* 2016). Analyses partitioning SNP-heritability by cell type-specific genomic annotations have shown enrichment of GWAS discoveries in trait relevant cells (Finucane *et al.* 2015).

### Examples of applications to psychiatric disorders

GREML SNP-heritability estimated for psychiatric disorders usually ranges from 15% to 30%, depending on the disease, and reported estimates are at most half of the estimates derived from family studies (Lee *et al.* 2012*a*, 2013; Anttila *et al.* 2016). SNP-heritability estimates of quantitative mental traits tend to be relatively low (<0.15), for example, a meta-analysis of the Big Five personality traits reported significant SNP-heritability estimates below 0.2 for all five traits (Lo *et al.* 2016). This is in line with another study of up to 300 000 people finding SNP-heritability estimates for subjective well-being, depressive symptoms and neuroticism in the same range (Okbay *et al.* 2016). Partitioning heritability by tissue-specific genomic annotations has identified the central nervous system as the most relevant tissue in the aetiology of schizophrenia and bipolar disorder (Finucane *et al.* 2015).

## Estimation of genetic correlation using genome-wide SNPs

Two traits are genetically correlated, if there is a correlation between the true effect sizes of SNPs affecting the two traits, or in other words, when, on-average, SNPs have directionally similar effects on two traits. Genetic correlation can reflect pleiotropy, which is defined as a genetic variant affecting both traits. However, while a non-null genetic correlation can imply pleiotropy is present at many SNPs, a genetic correlation of zero could arise when pleiotropy is common, but the direction of effects are uncorrelated across SNPs. Genetic correlations (*r*_G_) are of interest because they suggest a shared aetiology. However, misdiagnosis between two genetically uncorrelated diseases can generate significant estimates of *r*_G_ (Kendler, 1987; Wray *et al.* 2012). The availability of genetic marker data on disease case–control samples has allowed the interrogation of the genetic relationship between diseases, often for the first time, since traditional methods to estimate genetic correlation based on increased risk of a disease in relatives of those with another disease requires often unattainably large samples (Visscher *et al.* 2014).

Formally, genetic correlation is defined as genetic covariance between two traits, scaled by the genetic standard deviations of the two contributing traits. Methods used to estimate SNP heritability can be extended into a bivariate form to estimate *r*_G_. In order to estimate genetic correlation using GREML, individual-level genetic data and measurements on two phenotypes are required (from the same or from different individuals). The power of bivariate GREML analyses to detect *r*_G_ departing from 0 or from 1 depends on the population value of *r*_G_, the SNP-heritability of both traits, on the sample sizes, on whether the same or different samples are used for the two traits, and for disease traits, on the proportion of cases in the sample (Visscher *et al.* 2014). For example, for two diseases with lifetime prevalence of 1%, SNP-*h*^2^ of 0.2 and genetic correlation of 0.5, 5000 cases and 5000 controls for each disease are sufficient to have 89% power at type 1 error rate of 5% to detect a genetic correlation greater than 0, corresponding to a standard error of 0.06. The BOLT-LMM software is also capable of calculating *r*_G_ in a bivariate GREML analysis with shorter runtime (Loh *et al.* 2015). In contrast to heritability estimates, *r*_G_ estimates are scale independent (approximately) and hence scale transformation is not needed (Lee *et al.* 2012*b*).

If summary statistics on two or more traits are available, LDSC can be used to estimate *r*_G_ between them, albeit with higher standard errors than GREML. To make the application of LDSC for SNP-heritability and *r*_G_ estimation even more user-friendly, the LD Hub resource has been developed, which provides access to summary statistics from more than 200 different traits. LD Hub then calculates genetic correlation estimates between each of these traits and any additional traits for which the user provides summary statistics data (Zheng *et al.* 2017).

Estimation of *r*_G_ is most commonly applied to uncover genetic relationships between two different traits, but it can also be applied to detect heterogeneity in genetic effects between two different groups, for example a trait might be under different genetic control in men and in women or in old people and young people (i.e. G × E). It has also been applied to two data sets of the same disease, where *r*_G_ is expected to be one (Lee *et al.* 2013), to infer between-sample heterogeneity or two data sets of the same disease but of different ethnicity (De Candia *et al.* 2013). Calculating *r*_G_ across populations using LDSC is not straightforward, however, due to between population differences in both allele frequencies and LD structure. The program Popcorn addresses this problem and allows one to estimate the transethnic genetic correlation based on summary statistics and LD matrices from two populations (Brown *et al.* 2016).

### Examples of applications to psychiatric disorders

One of the first applications of the bivariate GREML method to disease traits was to estimate genetic correlations between psychiatric disorders, presenting evidence that most pairs of disorders result in estimates that are significantly different from zero (PGC cross disorder group (Lee *et al.* 2013)). From those initial estimates the high correlations between schizophrenia and bipolar disorder (~0.6) and between bipolar and MDD (0.5) were considered plausible given evidence from family studies; however, the high genetic correlation between schizophrenia and MDD was more surprising for the clinical community. In fact, close study of the literature from family studies (PGC Cross disorder group, 2013) showed that all three genetic correlation estimates were consistent with published increased risk to relatives (RR) of one disorder for individuals diagnosed with another. However, for the same genetic correlation the size of RR involving a very common disorder (~15% lifetime risk) such as MDD is much smaller than when both disorders are less common (<1% lifetime risk for both schizophrenia and bipolar disorder).

Genetic correlation can arise through misdiagnosis between two diseases. For example, those first presenting with clinical features consistent with a diagnosis of bipolar disorder can in the long-term receive a diagnosis of schizophrenia (and vice versa) (Joyce, 1984; Meyer & Meyer, 2009). However, it can be shown analytically that very high misclassification rate of 20% would be needed under no shared aetiology to result in *r*_G_ of ~0.6 estimated between schizophrenia and bipolar disorder (Wray *et al.* 2012). In contrast, a high genetic correlation between disorders would be consistent with some clinical presentations being difficult to classify.

**Fig. 2.**
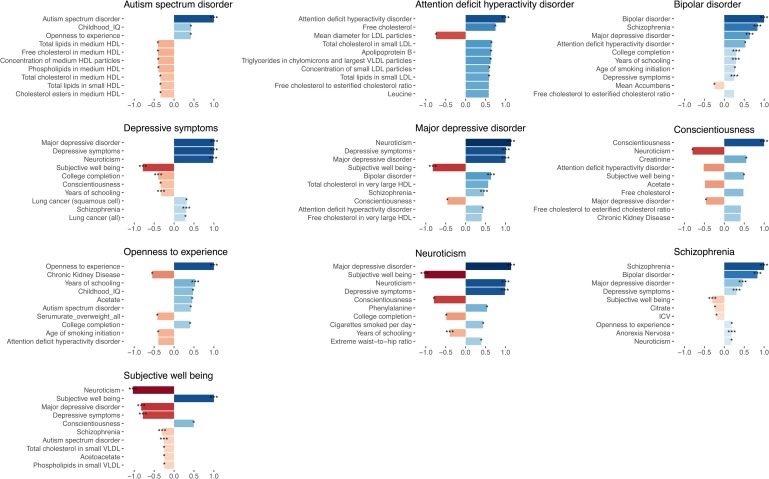
Genetic correlations between psychiatric disorders and traits, and almost 200 other traits. For each trait, the 10 traits with the highest absolute genetic correlations are shown. Colours indicate whether genetic correlations are positive or negative. One star indicates a genetic correlation *p* value <0.05. Three starts indicate a *p* value below the Bonferroni threshold of 2.81 × 10^−6^ for 17 766 tested trait pairs. Data obtained from LD Hub (Zheng *et al.* 2017).

LDSC has been applied to a full battery of GWAS summary statistics and report higher estimates of genetic correlations estimates between pairs of psychiatric disorders than between psychiatric- and non-psychiatric disorders (Bulik-Sullivan *et al.* 2015*a*). Some notable examples include a positive genetic correlation between schizophrenia and anorexia nervosa, and a positive genetic correlation between bipolar disorder and years of education. A study investigating genetic sharing between neurological and psychiatric traits found that among neurological disorders significant genetic correlations are rare, but that there is some overlap in genetic risk between migraine and MDD, and ADHD and Tourette syndrome (Anttila *et al.* 2016). On the other hand, genetic correlations between psychiatric traits and personality traits are more common. Figure 2 shows the top genetic correlations for psychiatric disorders and traits. Data obtained from LD Hub.

## Polygenic risk prediction

Estimates of SNP effects can be used to predict the genetic risk of individuals. Simple risk scores for each individual are calculated as the sum over all per SNP effects, where the per SNP effect is the allele count of the SNP for the individual multiplied by the effect size of the SNP (Wray *et al.* 2007; Purcell *et al.* 2009). Here the SNP effects come from a typically large and well-powered discovery (sometimes called training) data set. While the ultimate goal of genetic risk prediction is in applications where the phenotype has not yet been observed, in research applications the risk predictor is evaluated for individuals in a target (sometimes called validation or testing) data set where the phenotype has already been recorded, so that the efficacy of the predictor can be evaluated.

Screening of high-risk individuals for early intervention or prevention programs is a potential clinical application of polygenic risk prediction that at present is not widely used because of the low accuracy of genetic risk predictors (Chatterjee *et al.* 2016). However, there are applications of polygenic risk prediction in research where low prediction accuracy is less limiting; for example, it could be a cost-effective strategy to conduct follow-up studies in samples ascertained to be low or high for polygenic risk. The genetic predictor is evaluated against a measured phenotype in the target sample, which may or may not be the same phenotype from which the predictor was constructed. Generally, if the predicted and the measured trait are genetically correlated, then there should be positive prediction accuracy, given enough power in both data sets (Dudbridge, 2013).

In the usual implementation of polygenic risk scores, SNP effect sizes have been estimated from the standard, one SNP at a time GWAS analysis. The construction of polygenic risk scores is based on some decision about the proportion of SNPs to include in the predictor. As the discovery sample *p* value threshold becomes more lenient, the increased predictive power of including estimated effect sizes from more true positive associated SNPs is balanced by the inclusion of more false positives. The optimum proportion of SNPs to include depends on the (unknown) genetic architecture and size of the discovery sample. In the latest schizophrenia GWAS, the optimum *p* value threshold was identified as 0.05 (based on variance explained in out-of-sample prediction across many samples), although inclusion of all SNPs did not lower accuracy drastically (Ripke *et al.* 2014). Often a range of different *p* value thresholds are used to determine the best predictor, although this approach is prone to overfitting. Software such as PLINK (Purcell *et al.* 2007; Chang *et al.* 2015) implements basic polygenic risk scoring, while PRSice compares polygenic risk predictors using a large number of *p* value cutoffs to find the optimum threshold given the data (Euesden *et al.* 2015). In polygenic risk scoring, LD among SNPs is usually accounted for by applying LD-clumping, i.e. pruning of SNPs based on LD but with higher preference to SNPs with lower *p* values (usually simply termed ‘clumping’). Prediction accuracy can be improved by using methods that provide estimates of SNP effects that are conditional on all other SNPs, thereby directly taking SNP LD correlations into account (de Los Campos *et al.* 2013), such as Genomic Best Linear Unbiased Prediction (GBLUP) (Meuwissen *et al.* 2001), which is widely used in animal breeding. In GWAS, there are many more SNPs compared with individuals so effects sizes of each SNP are not estimable in a multiple regression model. In GBLUP, it is assumed that SNP effect sizes are drawn from a normal distribution and a shrinkage term, or penalty term, proportional to the trait heritability is introduced in the model. More complex models are BayesR (Moser *et al.* 2015) or LASSO (least absolute shrinkage and selection operator) (Abraham *et al.* 2013). These methods shrink the SNP effect estimates (i.e. the effect attributable to SNPs in LD with each other is shared between the correlated SNPs) and will ensure the predicted phenotypes are more accurate, and in LASSO and BayesR shrink a proportion of SNPs to zero. The gains in prediction accuracy are dependent on underlying genetic architecture (Moser *et al.* 2015).

If individual-level genotype data are not available, it is still possible to transform marginal SNP effects (standard GWAS summary statistics) into penalised, conditional SNP effects, by making use of an LD reference data set (Yang *et al.* 2012; Robinson *et al.* 2017). This is implemented in GCTA (Yang *et al.* 2011*a*) and in LDpred (Vilhjálmsson *et al.* 2015), which not only accounts for LD between SNPs, but also uses a Bayesian framework to adjust the SNP effects for traits where an infinitesimal model (all SNPs have some effect) is not the best fit to the data (e.g. autoimmune disorders). This is analogous to the selecting SNPs based on *p* value in standard polygenic risk prediction and can further improve accuracy.

Finally, GBLUP approaches can be extended to a multi-trait model, which can further improve prediction accuracy when phenotypes are genetically correlated, because measurements on each trait provide information on the genetic values of the other correlated traits. This approach has been implemented in the program MTG2 and has been shown to improve prediction accuracy for schizophrenia and bipolar disorder (Maier *et al.* 2015).

### Examples of applications to psychiatric disorders

Polygenic risk prediction is widely used in psychiatric genetics, not to infer an individual's case control status, but to gain a better understanding of disease aetiology. Polygenic risk prediction in applications relevant to psychiatry has been reviewed previously (Wray *et al.* 2014). Some more recent examples include schizophrenia polygenic risk scores calculated for community samples of individuals, which explain variation in creativity (Power *et al.* 2015) and cannabis use (Power *et al.* 2014). An association between schizophrenia polygenic risk scores and negative symptoms and anxiety disorder in adolescents gives reason to hope that these kind of studies can not only lead to a better understanding of disease aetiology, but may someday also contribute to early intervention programmes (Jones *et al.* 2016; Kendler, 2016). Polygenic risk prediction additionally provides a novel approach to studying gene – environment interactions (G × E). Traditional G × E studies, which test for an interaction effect between single genetic variants and an environmental exposure on disease risk, often suffered from low power caused by the small amount of variance explained by individual genetic loci. In contrast, interactions between a polygenic risk score and environmental exposure can be detected more easily, because polygenic risk scores explain more of the variance in disease risk than individual loci. This type of G × E study has been applied to investigate a potential interaction between a polygenic risk score for MDD and childhood trauma on the risk for MDD. Two independent studies found that both the polygenic risk score and exposure to childhood trauma increase the risk for MDD, but came to different conclusions about the nature of the interaction between the two: One study found a positive interaction, meaning that those with both exposure to childhood trauma and high polygenic risk scores are at the greatest risk of developing MDD (Peyrot *et al.* 2014), while another study found a negative interaction, where people with exposure to childhood trauma and low polygenic risk scores are at the highest risk (Mullins *et al.* 2016).

## Mendelian randomisation

MR analysis investigates the causal relationships between traits. It is a specific form of an instrumental variable analysis (Evans & Davey Smith, 2015), where the goal is to test the causal effect of an explanatory variable (exposure to a risk factor) on a dependent outcome variable (such as disease risk). In MR, genetic markers are used as the instrumental variables. The MR poster child is the application to blood lipid levels and myocardial infarction (Voight *et al.* 2012). Together with a number of randomised controlled trials (Keene *et al.* 2014), this application of MR has had major impact on drug development by providing evidence against a causal role of HDL and thus helped in the search for effective ways of preventing myocardial infarction, and demonstrates how evidence from an MR analysis could be used to circumvent costly randomised controlled trials.

**Fig. 3.**
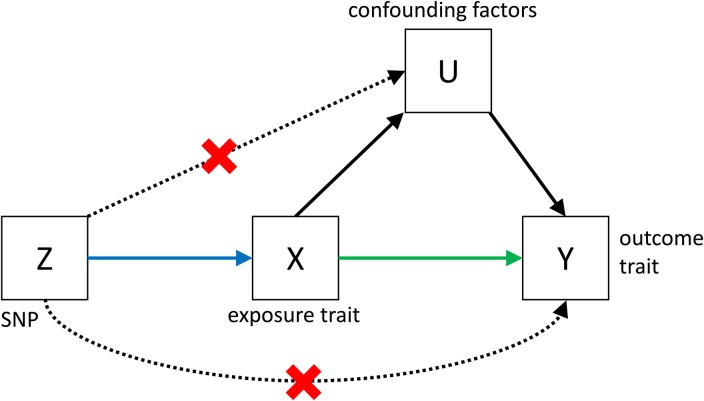
Causal pathways in an MR experiment. A causal effect of exposure (**X**) on outcome (**Y**) can be inferred if three core assumptions are met. These assumptions concern the genetic instrumental variable, **Z**, and state that: (i) Z has to be robustly associated with the exposure variable, (ii) Z cannot be related to any common causal factors of X and the outcome Y (these are labelled **U**), and (iii) Z may only be related to Y through X (Solovieff *et al.* 2013). The latter two assumptions can be summarised as the absence of pleiotropy for the instrumental variable.

Despite its great potential, MR is often limited by low power, and by the fact that it is very difficult to show that all the assumptions (see Fig. 3) that are necessary to infer causality are met. However, if MR is applied bidirectionally for trait pairs of approximately comparable power, and evidence for significant causality is detected in only one direction, then this can help to infer causality over pleiotropy. The power in MR studies is a function of the true causal association between exposure and outcome and of the variance explained by the instrumental variables (Brion *et al.* 2013). Since statistically significantly associated SNPs often only explain a small proportion of the genetic variance, for many pairs of traits, very large sample sizes are needed to achieve sufficient power to detect causal associations [see online calculator (Brion *et al.* 2013)].

In recent years, many improvements to the MR method have been developed. Of particular importance for polygenic traits is the extension from single-SNP instrumental variables to instrumental variables that comprise multiple SNPs (Evans *et al.* 2013). This may increase power since in polygenic traits individual SNPs are likely to be weak instruments. Two-sample MR is an extension that makes it possible to combine independent data for genotype – exposure and genotype – outcome in an MR experiment, thus allowing to investigate causal associations among all phenotypes for which well-powered GWAS data are available (Burgess *et al.* 2013). Further developments of MR include better ways to test some of the assumptions, modifications which allow the relaxation of the no-pleiotropy assumption, and improvements which increase the power to detect causal effects (Evans & Davey Smith, 2015). The web-based resource MR BASE has been developed to simplify the application of MR to test causality between a large number of traits and to compare different variations of the method (Hemani *et al.* 2016). Summary data for more than a thousand traits have been collected and can be tested for causal associations with data provided by the user.

### Examples of applications to psychiatric disorders

Several MR studies have investigated a potential causal influence of variables, which are known to be associated with psychiatric traits and diseases from observational studies. One study which looked at BMI as a potential risk factor concluded that there is no evidence of BMI being a causal influence on schizophrenia and bipolar disorder, but weak evidence of BMI conferring a higher risk of MDD. It was noted, however, that the BMI association suffers from low power caused by small sample sizes for MDD (Hartwig *et al.* 2016). C-Reactive protein (CRP) is a potential risk factor for psychiatric disorders with undisputed correlational association, but unclear causality. A study from 2016 surprisingly found a protective role of genetically elevated CRP levels on the risk for schizophrenia, as well as weak (nominally significant) evidence for a risk increasing effect on bipolar disorder as well as a range of other somatic traits (Prins *et al.* 2016). This is in contradiction with another 2016 study, which identified elevated CRP levels to confer an increased risk of schizophrenia (Inoshita *et al.* 2016). There is much debate on whether cannabis is a risk factor for psychosis or schizophrenia, or whether the association is due to reverse causation or due to a confounding factor (McGrath *et al.* 2010; Gage *et al.* 2016). Recently MR studies have provided evidence for a causal role of cannabis in the development of schizophrenia, but also for a reverse causation (Gage *et al.* 2017; Vaucher *et al.* 2017). Several other risk factors for schizophrenia, anxiety and depression have been investigated through MR with negative results (Bjørngaard *et al.* 2013; Gage *et al.* 2013; Taylor *et al.* 2016).

In summary, MR studies investigating risk factors for psychiatric disorders could in a few cases provide evidence for a risk increasing effect. As the power of GWAS with increasing sample sizes, there will be more robust SNP associations which can be used as instrumental variables. This will provide more evidence for whether the many negative results were just caused by low power or by the absence of a true causal association, but interpretation of causality needs to carefully consider confounding factors.

## Fine-mapping and gene prioritisation

LD between SNPs is both a blessing and a curse for GWAS. On one hand, it makes it possible to probe only a subset of all genetic variants yet still detect associations for a much larger set, either through tagging of non-genotyped SNPs by genotyped SNPs or through LD-based imputation to sequenced reference samples. On the other hand, it means that a detected association does not necessarily imply a causal role for the associated SNPs. Fine-mapping attempts to identify which out of a number of associated SNPs in a LD region have a causal role, and which are merely associated because they are in LD with causal SNPs (Sekar *et al.* 2016).

To better understand disease aetiology, it may be of interest to identify causal genes, rather than causal SNPs. In many cases the causal gene may simply be the gene closest to the most strongly associated SNP in a region, but this is not always so (Claussnitzer *et al.* 2015).

### Identifying causal SNPs

A range of different approaches have been developed for the fine-mapping of SNPs. Most of them use information on functional annotation of the genome and LD between SNPs, in addition to SNP association statistics on one or more diseases. An algorithm (PICS) utilising all these kinds of information has recently been applied to 21 autoimmune disorders and identified many putative causal variants by integrating information from different types of functional annotations, including epigenetic marks and gene expression information (Farh *et al.* 2015).

Fine-mapping methods can use LD information to either identify causal SNPs within a region that may not have the strongest association signal, but are located in a functional genomic element like an enhancer, or they can use LD information to identify multiple independently associated SNPs, by calculating the association signal conditionally on the association signal of neighbouring SNPs. Traditionally, this would require full genotype data on the trait of interest. However, it has been demonstrated that it is possible to borrow LD information from a reference genotype data set for a conditional analysis, making it possible to apply this approach to traits for which only summary statistics are available (Yang *et al.* 2012; GCTA-cojo). For this to work well, the LD structure in the reference genotype population should be a good approximation of the LD structure in the population on which the GWAS has been performed. Cross-ethnic genetic studies can aid fine mapping of disease loci, exploiting differences in allele frequency and LD (Morris, 2011), but application to psychiatric disorders has been limited by the dearth of non-European data sets.

Fine-mapping can benefit from data on multiple traits. When two traits share regions of significant genetic associations it can be investigated if they share causal loci at those shared regions, or if different loci drive the regional association in each trait. This has been investigated in a Bayesian framework using only summary statistics and resulted in the identification of 341 loci associated with more than one trait across 42 different phenotypes (Pickrell *et al.* 2016). SNPs associated with two traits form the basis of the previously discussed MR methods.

### Identifying causal genes

Genome-wide association studies suffer from a massive multiple-testing burden, owing to the large number of association tests between SNPs and phenotype. To minimise the number of false positive results, associations are usually required to be significant at a *p* value of 5 × 10^−8^ (Bonferroni correction of a million independent tests). Gene-based tests have a reduced multiple-testing burden (~20 000 independent tests) and give biological meaning to association results. In gene-based tests SNPs are aggregated into larger groups (Neale & Sham, 2004) assuming that SNPs exert their effect through nearby genes, which is not always true. The PrediXcan method refines this aggregation step by including external tissue specific eQTL data to predict gene expression levels based on SNP data (Gamazon *et al.* 2015). This has several advantages over conventional gene-based tests as it limits the multiple-testing burden by only using SNPs, which are known to affect gene expression, and the direction of effect of a SNP on expression levels is not lost when aggregating multiple SNPs. By using tissue-specific eQTL data, associations can be tested between a phenotype and expression changes in tissues relevant to the phenotype. PrediXcan has been used to identify genes which may play causal roles in amyloid deposition and cognitive changes in Alzheimer's disease (Hohman *et al.* 2017) and genes associated with Asthma (Ferreira *et al.* 2017). While PrediXcan requires individual-level genotype data, the extension MetaXcan requires only summary statistics and promises similar accuracy, if the right reference population is used for LD estimation (Barbeira *et al.* 2016). Transcriptome-wide association study (TWAS) is a summary statistics based method similar to MetaXcan, which differs in the algorithm used to predict expression (Gusev *et al.* 2016*a*). More recently, TWAS has been applied to detect pairs of traits with genetic correlations at the level of predicted expression (Mancuso *et al.* 2017). A current limiting factor in this and other expression-based methods is the quality of tissue-specific eQTL data. The previously mentioned methods prioritise genes based on predicted effects of SNPs on expression. This is in contrast to other methods, such as DEPICT, which use gene expression data to predict gene function and prioritise genes at specific loci based on the predicted function (Pers *et al.* 2015).

While an association of predicted gene expression and a phenotype is suggestive of a causal role for that gene, pleiotropy is an alternative explanation for this association. That is, the same SNPs could independently lead to expression changes in one gene and via a different route have an effect on the phenotype. The summary statistics-based MR (SMR) method (Zhu *et al.* 2016) attempts to distinguish between these two scenarios using eQTL SNPs as instrumental variables and gene expression as exposure variable. This method also applies a follow-up test (HEIDI), which identifies and excludes regions where multiple linked SNPs are independently associated with gene expression and phenotype, as a strategy to prioritise regions with evidence of a simple causal mechanism for functional follow-up. The method was applied to several complex human traits and has identified 126 putatively causal genes, of which 77 were not the closest gene to their respective top associated GWAS hit.

MetaXcan, TWAS and SMR use the same type of data to identify genes of interest. However, there are many subtle differences between the methods, which will likely lead to unique results for each method. To date, no systematic comparison of their relative performance has been published.

### Examples of applications to psychiatric disorders

PrediXcan has been applied to bipolar disorder, resulting in the identification of two genes, PTPRE and BBX, for which predicted increased expression in whole blood and the anterior cingulate cortex, respectively, was associated with increased risk of bipolar disorder (Shah *et al.* 2016). SMR has been applied to schizophrenia, highlighting two genes, SNX19 and NMRAL1, with a potentially causal influence (Zhu *et al.* 2016). The previous two examples have highlighted genes by using eQTL data, but the concept can be extended to account for the fact that chromatin modifications may mediate the association between genetic variants and eQTLs, and that splice-QTLs, rather than eQTLs, may underlie the genetic effect of a SNP. A TWAS study on schizophrenia has incorporated these ideas and has highlighted 157 genes, many of which were identified due to brain specific splice-QTLs (Gusev *et al.* 2016*b*).

## Detection of genetic heterogeneity

Most genetic studies are based on the assumption that individuals who exhibit similar symptoms or who have been diagnosed with the same disease are representatives of the same underlying biology defined by a common genetic architecture. Under a polygenic disease architecture, each individual is likely to have a unique combination of risk loci, but with each combination drawn from the pool of risk loci. Genetic heterogeneity occurs when individuals with the same clinical presentation have risk alleles drawn from independent (or perhaps correlated) sets of risk loci, and the genetic risk profile of one or more subgroups of cases departs from that of the rest. This may arise through misclassification of some cases, or through distinct aetiological pathways leading to the same diagnosis (Flint & Kendler, 2014; Jeste & Geschwind, 2014). The inherent phenotypic heterogeneity within psychiatry makes detection of genetic heterogeneity an appealing goal and identification of distinct pathways holds the promise of shedding light on disease aetiology. Furthermore, a biological basis for disease stratification could lead to more personalised treatments (Kapur *et al.* 2012).

Although the concept of identifying genetic sub-groups is intuitively appealing simulations suggest it is very difficult to find meaningful genetic groupings if each sub-group has a genetic architecture of a large number of loci with small effects. The large number of combinations of risk loci, their small effect sizes, the uncertainty about the size or even about the presence of genetically heterogeneous groups, and the challenging disentanglement from population stratification all contribute to the difficulty of this problem. As a result, even data sets comprising hundreds of thousands of individuals may not provide sufficient power for naïve approaches to detecting even the simplest scenarios of genetic heterogeneity (Han *et al.* 2016; Maier *et al.* unpublished results).

The BUHMBOX method (Han *et al.* 2016) frames the question of disease heterogeneity in a different way and sets out to test if two diseases that share a genetic basis (*r*_G_ > 0) are correlated because the shared genetic risk factors are present in the whole sample (pleiotropy) or are confined to only a subgroup of individuals (identifiable genetic sub-type) (Han *et al.* 2016). The BUHMBOX method investigates LD-independent risk loci for disease B in individuals diagnosed with disease A. If only a subgroup of individuals has a higher genetic risk for disease B, this will induce a correlation among the disease B risk loci, which would support the presence of genetic heterogeneity and not pleiotropy. This approach can demonstrate presence of a genetic sub-group without identifying which specific individuals diagnosed with disease A are genetically more similar to those with disease B. The method found evidence for heterogeneity among seronegative rheumatoid arthritis cases, suggesting that they may contain a significant proportion of seropositive cases. The power of the BUHMBOX method to detect heterogeneity depends on the number of cases, number of markers, risk allele frequency, odds ratio and proportion of disease A cases that are genetically disease B cases (heterogeneity proportion). For example, with 2000 cases and 2000 controls, a heterogeneity proportion of 0.2 and 50 risk loci, the power to detect heterogeneity at a significance threshold of 0.05 is 92% (Han *et al.* 2016).

Alternatively, genetic heterogeneity can be studied by first grouping individuals (for example, disease cases) based on non-genetic data and then testing for genetic heterogeneity between these disease subtypes. A recent method follows this approach by jointly modelling the probability for each SNP of whether its frequency differentiates cases and controls and/or differentiates disease subgroups. Applied to type 1 diabetes, this method suggests that cases with and without autoantibodies exhibit a different genetic architecture for type 1 diabetes disease risk (Liley *et al.* 2016).

### Examples of applications to psychiatric disorders

The BUHMBOX method was used to investigate the shared genetic basis between MDD and schizophrenia, and found no evidence that suggested that a subset of MDD cases was genetically more similar to schizophrenia cases, implying that the genetic correlation estimated between the disorders reflect pleiotropy (Han *et al.* 2016). Application of this method will become more interesting as sample sizes increase.

## Conclusions

For many psychiatric disorders, genetic factors explain more variation in disease risk in the population than any other known risk factors (Sullivan *et al.* 2012), but only recently has it become possible to resolve the overall familial genetic risk into individual risk factors at the DNA level. The evidence is now conclusive that psychiatric disorders, like many other common disease and disorders are highly polygenic underpinned by thousands of genetic loci, each of which contributes a small amount to the overall genetic risk. After a period in which many candidate gene studies have reported association results, which failed to replicate (Chanock *et al.* 2007), the hypothesis-free GWAS approach has established itself as the dominating paradigm to find associated genetic loci. With ever growing sample sizes, more and more SNPs surpass the stringent *p* value threshold for almost all investigated traits. However, it is also becoming clear that the bulk of genetic risk factors remains hidden among those loci that do not achieve genome-wide significance. Many of the methods presented in this review leverage the large amount of information that is harboured by genetic variants, regardless of whether or not they achieve significance. While the focus of some methods is on individual SNPs or genes, other methods aggregate over a potentially large number of loci to answer questions such as ‘What is the combined genetic effect of all measurable SNPs on phenotypic variance?’, ‘Do these traits have a shared genetic aetiology?’ or ‘Do these traits causally influence one another?’. One thing that all of these methods have in common is that their utility crucially depends on the power to detect an association, which in turn depends on sample size. Larger sample sizes lead to a higher computational burden, but for most analytical questions, which have been presented here, there are methods which can utilise summary statistics and thus drastically reduce runtime and memory requirements.

The literature on new methods is ever-growing and while we have tried to present an overview of key methods to help navigation of this complex field, it is difficult to be fully exhaustive. In particular, pathway analyses are important application of GWAS summary statistics, but these methods have been reviewed recently (Wang *et al.* 2010) and so were not considered here. We have illustrated the methods with some example applications; however, we expect the full potential of the data will only be revealed in coming years when studies with half a million or more people will be widely available. A key issue for the field is to develop cost-effective strategies to capture larger sample sizes with both DNA samples and phenotypic data as these are needed to evaluate the extent to which genetic data can explain phenotypic heterogeneity and to fulfil the potential of more personalised medicine.
